# Systematic Review of Tinea Nigra: A Clinical Approach

**DOI:** 10.3390/jof11040287

**Published:** 2025-04-06

**Authors:** Miguel Ángel Sánchez-Romero, José Ramón García-Lira, Norma Olivia de la O-Escamilla, Dulce Melissa Martínez-Tellez, Elizabeth Esther Cortés-Salazar, Adriana María Valencia-Herrera, Mirna Eréndira Toledo-Bahena, Carlos Alfredo Mena-Cedillos, Sonia Toussaint-Caire, Marcela Salazar-García, Alexandro Bonifaz

**Affiliations:** 1 Dermatology Department, Federico Gomez Children’s Hospital of Mexico, National Institute of Health, Cuauhtemoc, Mexico City 06720, Mexico; masromero98@hotmail.com (M.Á.S.-R.); ramongarcialira@gmail.com (J.R.G.-L.); normaldelao_16@hotmail.com (N.O.d.l.O.-E.); dra.martineztellez@gmail.com (D.M.M.-T.); elicortsala@gmail.com (E.E.C.-S.); mirnatoledo@gmail.com (M.E.T.-B.); camenac@gmail.com (C.A.M.-C.); 2 Dermatopathology Department, Hospital General Dr. Manuel Gea Gonzalez, Tlalpan, Mexico City 4800, Mexico; reportestouss@hotmail.com; 3 Biomedical Research Department, Federico Gomez Children’s Hospital of Mexico, National Institute of Health, Cuauhtemoc, Mexico City 06720, Mexico; msalazar.investigacion@gmail.com; 4 Mycology Department, Hospital General de Mexico Dr. Eduardo Liceaga, Mexico City 06720, Mexico; a_bonifaz@yahoo.com.mx

**Keywords:** tinea nigra, *Hortaea werneckii*, superficial mycosis, hyperpigmented lesions

## Abstract

Tinea nigra (TN) is a superficial fungal infection caused by the melanized fungus *Hortaea werneckii*, characterized by irregular dark patches, typically on the palms. This systematic review aims to evaluate the epidemiology, demographic characteristics, lesion distribution, diagnostic approaches, causative agents, and treatment outcomes of TN. The PubMed and MEDLINE databases were systematically searched using relevant keywords from January 1990 to January 2025, yielding a total of 102 cases across 42 studies. TN is more prevalent in tropical regions, with a higher incidence in the Americas (64 cases), particularly Mexico (23 cases), Brazil (17 cases), and Cuba (8 cases). The infection is more commonly observed in females (58 cases) than males (44 cases), with a mean patient age of 16.7 years (SD ± 13.58). The most frequently affected anatomical site is the palm, with 41 cases on the left palm, 34 on the right, and 5 involving both palms. Other affected sites include the soles and interdigital areas. Diagnosis typically involves direct microscopic examination using potassium hydroxide (KOH) preparation, which was performed in all cases, while cultures were conducted in 96 cases and dermoscopy was used in 14 cases. *Hortaea werneckii* was the predominant species isolated (74 cases), followed by *Exophiala werneckii* (14 cases), *Pullularia werneckii* (4 cases), and *Aureobasidium melanogenum* (3 cases). Recently, a new etiologic agent, *Cyphellophora ludoviensis*, was reported, among others. Over 25 treatment modalities were reported, with topical therapies being the most common. Whitfield’s ointment was used in 12 cases, followed by ketoconazole 2% cream (11 cases), and terbinafine 1% cream and isoconazole 1% cream (10 cases each). Spontaneous resolution occurred in two cases. The average treatment duration across all modalities was 4 weeks, with a 100% resolution rate. This systematic review emphasizes the importance of understanding TN’s clinical presentation, diagnostic techniques, and therapeutic strategies to optimize patient care and guide future research on this relatively uncommon fungal infection.

## 1. Introduction

Tinea nigra (TN) is a rare superficial mycosis caused by the melanized fungus *Hortaea werneckii*, affecting the stratum corneum of the skin. It most commonly presents as a solitary, well-defined dark brown or black patch on the palms, although it can also appear on other body sites such as the soles, neck, or trunk, especially in a bilateral form [1,2,3]. TN is usually asymptomatic and may exhibit fine scaling.

Diagnosis is based on clinical evaluation, supported by dermoscopy, and confirmed through direct mycological examination using potassium hydroxide (KOH) and/or mycological culture. Histopathological examination is only required to exclude malignant melanocytic lesions.

TN is more prevalent in tropical and subtropical regions, accounting for 0.085% to 0.1% of reported mycoses in Latin American centers. In non-endemic areas, it is typically associated with travel history [4,5]. The condition predominantly affects individuals under 20 years of age, with a slight female predominance. Hyperhidrosis is a major risk factor, as the salinity promote fungal growth. The infection is likely acquired from sandy soils with microabrasions [5,6].

TN generally responds well to topical antifungals. Spontaneous resolution, though documented in some cases, remains rare [7,8,9].

### 1.1. Definition

**Figure 1:**
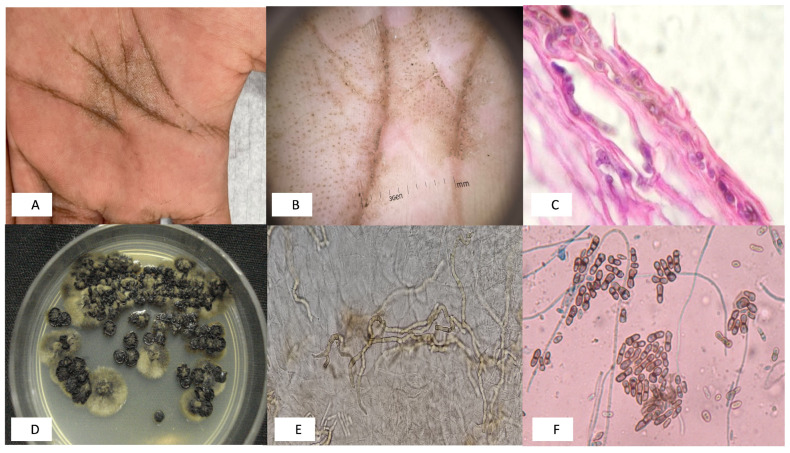
(**A**) Clinical photograph of tinea nigra on the palm. (**B**) Dermoscopic image. (**C**) Skin biopsy stained with hematoxylin and eosin (100× magnification) (Courtesy of Dr. Clemento Moreno-Collado, CDMX, Mexico). (**D**) Fungal culture, in yeast-mold phase. (**E**) Direct examination with KOH with dark hyphae (Courtesy of Dr. Fernando Gómez-Daza, Venezuela). (**F**) Fungal microscopy of *H. werneckii* with typical annelloconidia (Cotton blue, 40×).

TN is a rare chronic superficial mycosis, whose causal agent is dematiaceous or the melanized fungus *Hortaea werneckii*, confined to the stratum corneum of the skin (Figure 1). It presents as a well-defined, typically rounded spot, dark brown or blackish in color, potentially covered by fine scales, and is generally asymptomatic [10,11,12,13].

### 1.2. Synonymy

TN, superficial phaeohyphomycosis, phaeoanelomycosis, *tinea nigra palmaris*, *keratomycosis nigricans*, and others [1,14].

### 1.3. Historical Background

First described in 1891 by Alexandre Cerqueira in Bahia, Brazil, it was initially termed “*keratomycosis nigricans palmaris*”. Although he did not publish his findings, his son incorporated them into his thesis. In 1905, Aldo Castellani associated TN with a mycosis initially attributed to *Microsporum mansoni*, later renamed *Cladosporium mansoni* by Mason. In 1911, Castellani described the first case in a European who traveled to Myanmar and Sri Lanka. Over the decades, the fungus was called *Exophiala werneckii* (1970) by Von Arx and *Hortaea werneckii* (1984) by Nishimura and Miyaji. In 1985, McGinnis and Schell demonstrated that *C. mansoni* and *C. werneckii* were the same species, renaming it *Phaeoannellomyces werneckii.* The current designation is *H. werneckii*, recognizing Horta as the first author. Confusion arises from its polymorphic nature, appearing as both black yeast and mold. In 1992, Zalar and de Hoog highlighted its halophilic and halotolerant properties, explaining why most cases occur in tropical and subtropical regions. In 2008, Bonifaz and colleagues, by sequencing rDNA in Mexican cases, contributed to a more precise identification of this microorganism [12,13,14,15,16].

### 1.4. Causal Agent

The primary causative agent of TN is *H. werneckii* (formerly known as *Exophiala werneckii*, *Cladosporium werneckii*, or *Phaeoannellomyces werneckii*), a pleomorphic dematiaceous fungus that initially grows as black yeast and later transforms into mold [1]. Two other fungal microorganisms have been linked: *Cladosporium castellani* (previously called *Stenella araguata*) and *Phoma hibernica.* This fungus exhibits significant osmoadaptation capabilities, attributed to intracellular glycerol and erythritol concentration, coupled with melanization of the cell wall, enhancing osmolyte retention. It can thrive in a broader salinity range than most known organisms. Due to its characteristics and molecular studies of mitochondrial DNA (mtDNA), its probable aquatic origin has been proposed [17,18,19]. Other dematiaceous fungi such as *Stenella araguata* have been associated with TN cases, and *Curvularia species*, particularly *Curvularia lunata*, described in decomposing plants and soil, have been identified in isolated cases [20,21].

### 1.5. Pathogenesis

The transmission of TN occurs through direct contact of micro traumatized skin and surfaces with its natural environment, characterized by high humidity and salinity. For instance, contact with beach sand or coastal areas with hands or bare feet is a common route, and person-to-person transmission is not considered. Recurrences are typically attributed to exposure rather than interhuman transmission. The skin probably suffers microtrauma that inoculates the fungus and adheres it to the surface of the corneal layer [22,23,24].

Upon entering the stratum corneum through microtrauma, the lamellar arrangement and characteristics of corneocytes and the extracellular matrix often prevent the fungus’s proliferation, leaving it as a commensal agent. Adhesion to the skin can be explained by hydrophobic interactions or the production of extracellular polysaccharides. The fungus persists through the assimilation of lipid products [23,24,25].

The palms and soles, characterized by a high concentration of sweat glands, are the most frequently affected areas. The primary risk factor is palmar or plantar hyperhidrosis, as the conditions of humidity and hypersalinity facilitate the development of TN. No immunosuppressive conditions or genetic factors have been identified as risk factors [25,26,27,28].

### 1.6. Epidemiology

TN is commonly observed in women, with a ratio of 2 to 1. Cases have been reported across all age groups, from newborns to the elderly, but there is a higher incidence in children and young adults. The disease is classically associated with tropical and subtropical climates, prevalent in Central and South America, Asia, the Polynesian region, and Africa [14,15,16]. In Latin American centers, it represents 0.085% to 0.1% of reported mycoses in hospital settings. Cases reported in non-endemic areas like Europe and North America are often linked to a history of travel, with up to 50% of patients having traveled to representative regions [15].

*H. werneckii* has been isolated not only in regions with the mentioned climate but also in puddles, desalinators, household dust, guinea pig burrows, and even on the surface of leaves of Chinese medicinal plants [24]. The incubation period of the fungus is not entirely clear, but symptoms typically manifest 15 to 20 days after travel [15]. The duration from symptom onset to diagnosis and cure varies between 1 to 18 months [16]. The prevalence in warm environments is attributed to the halotolerant and halophilic properties of *H. werneckii*, allowing it to thrive in humid and hypersaline conditions [15,16].

### 1.7. Clinical Presentation

TN manifests as irregularly shaped plaques composed of hyperpigmented patches, ranging in color from light brown to dark brown or black, with well-defined borders. Some cases exhibit more pigmentation and fine scales on the surface, without erythema. Lesions grow centrifugally. An atypical morphology with a mottled or “salt and pepper” pattern has been described in some cases. The palmar region is the most common site, usually unilateral, with a left palm predominance; in sporadic cases, discreet erythema occurs; these cases are related to little pruritus. However, it can also be bilateral, affecting the soles in 10 to 20% of cases, and, less frequently, other areas such as fingers, interdigital spaces, arms, legs, neck, and trunk. The evolution is typically chronic and asymptomatic, with only a minority of patients reporting mild pruritus [18,19,20,21].

### 1.8. Diagnosis

Accurate diagnosis of TN requires a thorough clinical history and a comprehensive physical examination. In cases of diagnostic uncertainty, various confirmatory or ancillary diagnostic tools should be employed to avoid unnecessary biopsies (Table 1) [21,22,23].

The clinical diagnosis must be confirmed by identifying the causal agent through direct mycological observation of lesion scales. This is achieved by applying 10% potassium hydroxide preparation, followed by microscopic examination, or by culturing on Sabouraud dextrose agar at room temperature. Under the microscope, mycelia composed of short, tortuous dematiaceous hyphae (melanin) are observed. The use of chlorazole black solution is not recommended, because it gives a dark color to any fungal element. KOH alone is preferable, which allow us to recognize dark or ocher-colored (amber) hyphae. After 10 days of growth on Sabouraud dextrose agar, the culture exhibits restricted, black, moist-looking colonies initially resembling yeast, which later become filamentous with wide, densely septate, thick-walled, brown hyphae [29,30,31].

Dermoscopy, a non-invasive diagnostic tool, has proven effective in identifying TN. Brown pigmented spicules forming a homogeneous reticular pattern, distinct from melanocytic lesions, are commonly observed. Dermoscopic patterns may occasionally resemble melanocytic lesions, requiring further investigation. Fine, faint brown spicules arranged in a parallel ridge pattern have been described as characteristic of TN. This dermoscopic feature, also referred to as the arborescent pattern, aids in distinguishing TN from melanoma [30,31,32]. In cases where previous diagnostic methods are inconclusive, particularly when melanocytic lesions are suspected, a skin biopsy may be performed.

In vivo reflectance confocal microscopy is a useful tool for patients with potential malignant melanocytic lesions, providing a rapid, non-invasive, real-time examination. This technique reveals elongated structures with a mottled or round appearance in the stratum corneum, corresponding to septate hyphae and blastoconidia, respectively. No inflammation, pagetoid cells, melanocyte nests, or atypical cells are reported in the surrounding tissue [27].

Additionally, scanning electron microscopy has been described, involving the analysis of epidermal samples obtained through superficial shaving of a TN lesion. The external skin fragment shows an epidermis with corneocytes, hyphae, and elimination of fungal elements, while the internal surface displays hyphal aggregates forming fungal colonies between keratinocytes. These findings align with dermoscopic observations, where hyphal aggregates between keratinocytes correspond to brown spicules [33,34,35].

### 1.9. Differential Diagnose

TN presents challenges in differential diagnosis due to its resemblance to various melanocytic lesions and other skin conditions. It may be mistaken for junctional melanocytic nevi, nevus spilus, malignant melanoma, or conditions like post-inflammatory hyperpigmentation, pigmented fixed erythema, Addison’s disease pigmentation, and melanosis from secondary syphilis or chemical exposure [34,35,36]. Furthermore, it is crucial to exclude the possibility of exogenous pigment deposits, such as henna, silver nitrate, potassium permanganate, plants, or fruits [36,37].

### 1.10. Treatment

Management of this superficial mycosis involves the use of topical keratolytics and/or antifungals [36,37,38]. While some of the literature suggests the potential for spontaneous remission [2,3], both topical and systemic therapies have been explored, with resolution generally expected within two to three weeks. The choice of treatment depends on factors such as the extent of the infection, prior treatment responses, and patient-specific considerations.

Topical imidazoles, including miconazole, ketoconazole, econazole, oxiconazole, and bifonazole, are typically applied twice daily until clinical resolution is achieved. These agents work by inhibiting the synthesis of ergosterol, an essential component of the fungal cell membrane. Oral triazoles, such as itraconazole, voriconazole, and posaconazole, are reserved for more extensive or resistant cases and function similarly by targeting ergosterol biosynthesis. Allylamines like terbinafine, available in both topical and systemic formulations, act by inhibiting squalene epoxidase, another key in fungal cell membrane production.

Whitfield’s ointment, which contains 3% salicylic acid and 6% benzoic acid, has demonstrated efficacy due to its keratolytic properties and antifungal activity. Additional keratolytic agents, such as salicylic acid alone or urea-based preparations, may be used to enhance the penetration of antifungals, although their standalone efficacy is limited.

The emergence of antifungal resistance is a concern in clinical practice, particularly with triazoles. Therefore, treatment selection may be influenced by known local resistance patterns. For refractory or severe cases, systemic antifungals typically used for invasive mycoses, such as amphotericin B and caspofungin, have shown some effectiveness [39,40]. However, their use is generally limited due to potential toxicity.

Recurrence is not uncommon, and preventive measures such as maintaining dry skin, using antifungal powders, and addressing predisposing factors (e.g., hyperhidrosis) are recommended to reduce the risk.

### 1.11. Prognosis

TN typically responds well to topical treatment and usually does not recur after completion. However, if there is a lack of response, it is essential to assess the patient’s adherence to the treatment regimen. The persistence of risk factors may predispose individuals to reinfection. Spontaneous remission of TN has also been documented in the literature.

## 2. Methods

A comprehensive search was conducted on 29 January 2025, utilizing databases such as PubMed and SCOPUS between January 1990 and January 2025. The keywords “tinea nigra” OR “superficial mycosis” were employed without imposing restrictions on time or language. This systematic search yielded a total of 691 articles. Inclusion criteria encompassed articles featuring patients diagnosed with TN, providing a thorough clinical presentation, time to diagnosis, and details on KOH microscopy and mycological culture, as well as information on the administered treatment and its duration.

**Figure 2:**
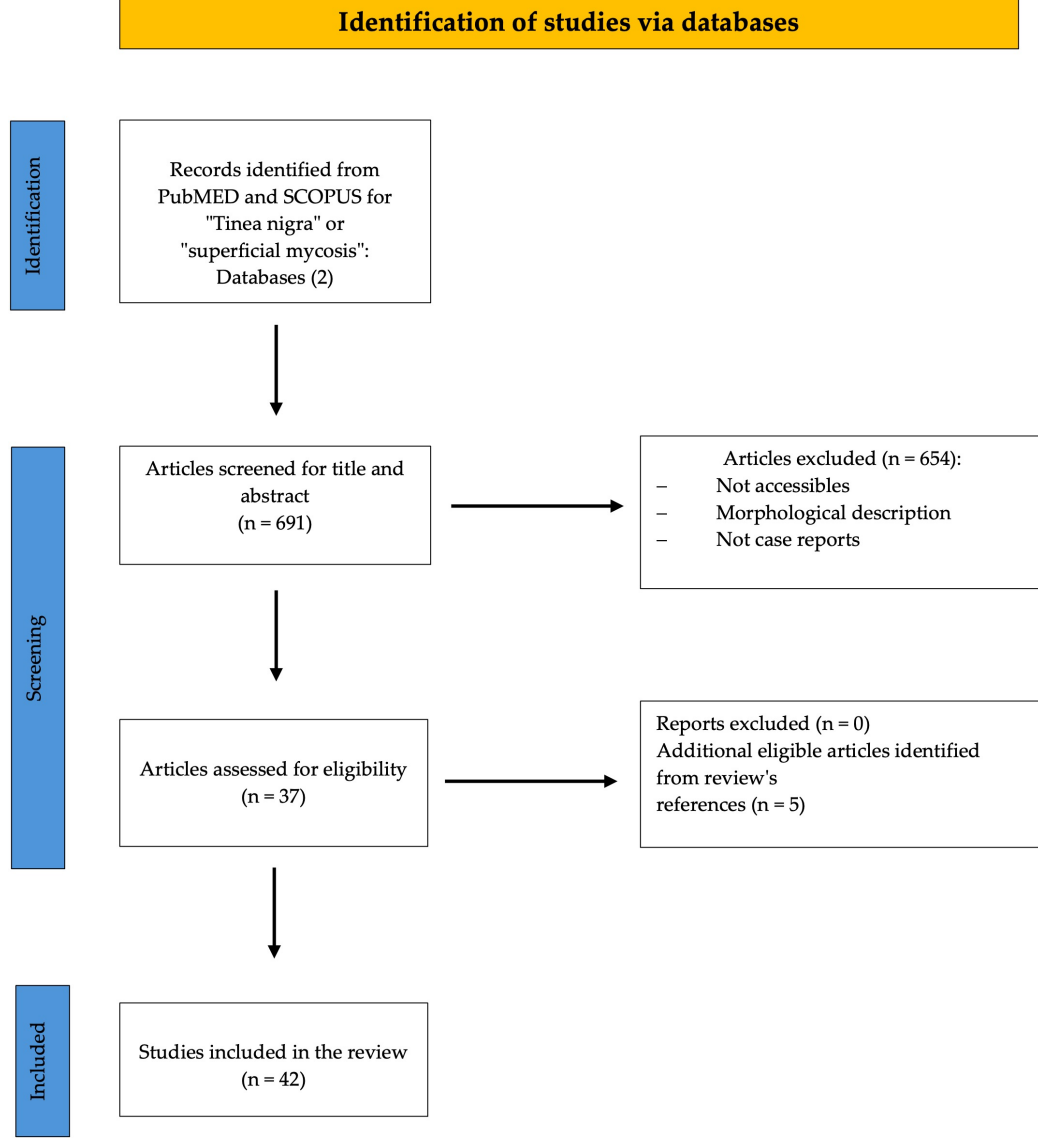
PRISMA flowchart outlining and presenting systematic review methodology.

Of the initial search results, 654 articles were excluded as they did not meet the specified inclusion criteria. Additionally, three more eligible articles were identified through references in other reviews. Consequently, the final dataset comprised 42 articles. To ensure a rigorous review process, all articles, including titles, abstracts, and full texts, underwent independent evaluation by two authors (M.A.S.-R. and E.E.C.-S.). The systematic review methodology is delineated in Figure 2, presented in the form of a flowchart.

## 3. Results

Upon analyzing the available literature, a total of 102 cases of TN were identified. Demographic data, including sex, age, lesion topography, diagnostic methods (dermoscopy, KOH), isolated microorganisms by culture, and treatment approaches, were recorded.

The distribution between male and female patients was 44 and 58 cases, respectively. The mean age of the patients was 16.7 years (SD ± 13.58). The most frequently affected clinical topography was the palm, with 41 cases on the left palm, followed by 34 cases on the right palm, and 5 cases with lesions on both palms. Other reported topographies included the right sole (five cases), bilateral plantar involvement (four cases), and nuchal/interdigital involvement (two cases each). Notably, during the literature search, a rare case was found where a patient on peritoneal dialysis developed peritonitis due to *Hortaea werneckii,* suggesting potential dissemination in immunocompromised individuals.

Epidemiological analysis revealed a predominant occurrence in the American region (64 cases), specifically in Mexico (23 cases), Brazil (17 cases), Cuba (8 cases), and others. The Asian continent reported fourteen cases, while Europe had six cases, and the Australian region reported only two cases. The African continent did not report any cases of TN.

For diagnostic purposes, culture was performed in 98 cases, supported by KOH examination in all cases and dermoscopy in 14 cases. Dermoscopy played a crucial role in enhancing diagnostic accuracy by enabling detailed visualization of specific skin lesion characteristics. A notable example is the recent work by Saikawa et al. [41], in which a dermoscopic pattern suggested melanoma, but a comprehensive mycological study ultimately defined the case. This highlights the importance of integrating multiple diagnostic modalities to rule out melanocytic lesions.

The most frequently isolated microorganism from cultures was *Hortaea werneckii* (seventy-four cases), followed by *Exophiala werneckii* (fourteen cases), *Pullularia werneckii* (four cases), *Aureobasidium melanogenum* (three cases), *Stenella araguata* (two cases), *Curvularia lunata* (one case), *Microsporum canis* (one case), and *Phaeoannellomyces werneckii* (one case). Recently, a new etiologic agent has been reported: *Cyphellophora ludoviensis* [42].

Concerning treatment, over 25 formulations were utilized, with the most common being “Whitfield Ointment” (12 cases), followed by “Ketoconazole 2% cream” (11 cases), and Terbinafine 1% cream, along with “Isoconazole 1% cream” (both with 10 cases). Additional therapies included clotrimazole 1% cream, ciclopirox 1% cream, itraconazole 200 mg PO, butenafine 1%, urea 15% cream, miconazole cream 2%, tolnaftate cream 1%, sertaconazole 2%, griseofulvin 250 mg PO, topical naftifine, griseofulvin cream, sulfur soap, imidazole cream, and oxiconazole cream, among others.

Notably, two cases reported spontaneous healing. The average treatment duration across all formulations was 4 weeks, with a 100% resolution rate.

## 4. Discussion

The analysis of TN cases highlights key epidemiological, clinical, and therapeutic aspects of this superficial fungal infection. TN is predominantly reported in tropical regions, particularly in the Americas, with Mexico, Brazil, and Cuba accounting for most cases [14,15,16]. This distribution suggests that environmental factors, such as high humidity and temperature, play a crucial role in its pathogenesis.

The absence of reported cases in Africa, despite favorable climatic conditions for fungal growth, raises concerns about potential underdiagnosis or underreporting [15]. Given the limited literature on TN, accurate case documentation is essential to enhance epidemiological understanding and guide effective management strategies.

Demographically, the higher incidence in females and younger individuals (with a mean age of 16.7 years) aligns with previous observations in other superficial fungal infections, which may be linked to increased exposure to environmental sources of infection, such as contaminated water or soil. The higher prevalence in children and young adults could also be associated with more frequent engagement in outdoor activities or closer contact with potentially contaminated surfaces [14,15].

Clinically, the most common presentation involves lesions on the palms, which may reflect the higher likelihood of direct contact with contaminated surfaces. The presence of cases involving the soles and interdigital areas indicates that TN can also manifest in less typical sites, suggesting that clinicians should consider TN in the differential diagnosis of hyperpigmented lesions in various anatomical locations, particularly in endemic regions [18,19,20].

The diagnostic methods employed in these cases underscore the utility of potassium hydroxide (KOH) preparation and culture as standard diagnostic tools, with dermoscopy serving as a valuable adjunct in visualizing characteristic lesion features [21,22]. The predominance of *Hortaea werneckii* as the primary pathogen supports its well-established role in TN, though the identification of other microorganisms, such as *E. werneckii* and *Aureobasidium melanogenum*, highlights the potential for misidentification or co-infection in certain instances [22,23].

It is also important to note that most reported cases originate from developing countries, where access to advanced molecular or genetic studies is limited. However, given TN’s benign nature, such studies are not essential for diagnosis or treatment. Nonetheless, future research incorporating molecular techniques could provide valuable insights into its epidemiology and pathogen diversity.

Treatment outcomes demonstrate a high rate of success with topical therapies, particularly Whitfield’s ointment and ketoconazole cream, which were among the most frequently used options [36,37]. The average treatment duration of four weeks aligns with the expected course for superficial mycoses, and the 100% resolution rate indicates that TN is highly responsive to appropriate topical treatment, likely due its superficial nature and high susceptibility to antifungal agents.

The spontaneous resolution reported in two cases suggests that, in some patients—particularly those with limited lesions or robust immune responses—observation may be a reasonable initial approach. However, antifungal therapy remains the standard of care to ensure complete resolution and reduce the risk of recurrence [37,38].

One of the main limitations of this review lies in the extensive period covered by the literature included, as well as the changes in nomenclature over time, which may impact the interpretation of the data.

Future research should aim to explore the reasons behind geographic disparities in TN cases and investigate the genetic or environmental factors that may predispose certain populations to infection.

## 5. Conclusions

TN is a rare superficial mycosis caused by *Hortaea werneckii,* primarily affecting the palms of individuals in tropical regions. Diagnosis relies on clinical evaluation, KOH preparation, culture, and dermoscopy. Topical antifungals, such as Whitfield’s ointment and ketoconazole, ensure effective treatment.

Understanding its epidemiology and clinical features is crucial, especially in non-endemic regions where travel history aids diagnosis. The limited literature highlights the need for further research.

## Figures and Tables

**Table 1:** These diagnostic tools can help to diagnose tinea nigra and differentiate it from other skin conditions with similar presentations.

Diagnostic Tool	Description
Clinical examination	Hyperpigmented, irregular, well-defined, asymptomatic macules on one of the palms or soles.
Dermoscopy	“Brown spicules” forming a reticulated patch or arranged in a parallel ridge pattern with one homogeneous color. Also called the “arborescent pattern”.
Histopathology	Mild acanthosis with thickening of the stratum corneum and the presence of pigmented, short or ramified hyphae and sometimes with blastoconidia.
Direct examination with KOH	Brown or ocher septate hyphae with thick walls and sometimes with blastoconidia.
Fungal culture	Dark color (greenish black) Initially (3–5 days) creamy yeast-like colony, later becoming moldy.
Fungal microscopy	Hyphae plus dark anelloconidia with septum.
Reflectance confocal microscopy	Multiple hyphae with globose, bottle-shaped conidiogenous cells.
Scanning electron microscopy	Multiple shiny, elongated, or round structures in the stratum corneum.

## Data Availability

The original contributions presented in the study are included in the article, further inquiries can be directed to the corresponding author.
